# 1213. Vaccine Uptake Amongst Participants in the North Carolina COVID-19 Community Research Partnership Who Were Initially Receptive or Hesitant to Receive a COVID-19 Vaccine

**DOI:** 10.1093/ofid/ofab466.1405

**Published:** 2021-12-04

**Authors:** Iqra Munawar, Austin L Seals, John W Sanders, David M Herrington, Thomas F Wierzba

**Affiliations:** 1 Wake Forest School of Medicine, Winston-Salem, North Carolina; 2 Wake Forest Baptist Health, Winston-Salem, North Carolina; 3 Wake Forest University School of Medicine, Winston Salem, North Carolina

## Abstract

**Background:**

Public health officials are concerned that adults may refuse to be vaccinated with an approved COVID-19 vaccine thereby limiting the community health benefit. Here, we studied the self-reported intent to be vaccinated of persons in North Carolina (NC) and then measured whether they did or did not get vaccinated.

**Methods:**

The Community COVID-19 Research Partnership (CCRP) is a large prospective study exploring COVID-19 epidemiology and sequelae in participants of several mid-Atlantic and Southern States. All participants complete an online daily survey where they are asked questions about COVID-like symptoms, infections, and their vaccination status. In addition to the daily survey, in December 2020, we implemented a short online cross-sectional survey questioning NC participants on whether they intended to be vaccinated. After completing the cross-sectional survey, we used daily survey data through 15 May 2021 to see if participants reported receiving vaccine. Unvaccinated participants who did not complete the daily survey 30 days or more prior to 15 May 2021 were excluded.

**Results:**

18,874 participants completed the cross-sectional survey and reported vaccination status. Of these participants, 90% were white, 68% were female, 26% were healthcare workers, and 2% self-reported COVID-19 diagnosis The median age was 54 years (IQR: 41 – 65). 79%, 13%, 9%, and 2% answered yes, unsure, no, and prefer not to answer, respectively, about intention to be vaccinated (Table). 99% of the participants who intended to receive the COVID-19 vaccine reporting being vaccinated. Those who were unsure or intended not to get vaccinated had vaccination rates of 80% and 53%, respectively. 78% of the participants who preferred not to answer were vaccinated.

**Table:**

Vaccine intent versus vaccine status – COVID-19 Community Research Partnership, North Carolina, December 2020 – May 2021

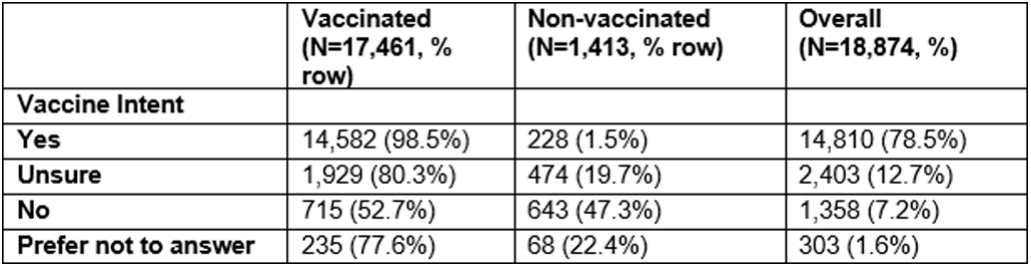

**Conclusion:**

More than three-quarters of NC participants intended to get vaccinated and by mid-May 2021, the vast majority had received at least one dose. Similarly, those who were unsure or preferred not to say were mostly vaccinated. Even among those who reported they would not get vaccine in January, more than half had received vaccine by May. The nature of our sample makes it difficult to generalize results to the population of NC; nevertheless, further investigation as to the causes of the shift in attitudes is warranted.

**Disclosures:**

**All Authors**: No reported disclosures

